# Usefulness and safety of remimazolam in upper gastrointestinal endoscopy: A comparative study between elderly and non‐elderly patients

**DOI:** 10.1002/deo2.70057

**Published:** 2025-01-16

**Authors:** Ryoji Ichijima, Hisatomo Ikehara, Daisuke Yamaguchi, Yasuhiko Nagata, Kanako Ogura, Mitsuru Esaki, Yosuke Minoda, Hiroyuki Ono, Yuki Maeda, Shinsuke Kiriyama, Tetsuya Sumiyoshi, Yuichi Kanmura

**Affiliations:** ^1^ Division of Gastroenterology and Hepatology, Department of Medicine Nihon University School of Medicine Tokyo Japan; ^2^ Department of Gastroenterology Saiseikai Kawaguchi General Hospital Saitama Japan; ^3^ Department of Gastroenterology Kiriyama Clinic Gunma Japan; ^4^ Department of Gastroenterology Kitasato University School of Medicine Kanagawa Japan; ^5^ Department of Gastroenterology National Hospital Organization Ureshino Medical Center Saga Japan; ^6^ Division of Gastroenterology Department of Internal Medicine Faculty of Medicine Saga University Saga Japan; ^7^ Department of Gastroenterology Nagata Surgery and Gastroenterological Clinic Tokyo Japan; ^8^ Department of Medicine and Bioregulatory Science Graduate School of Medical Sciences Kyushu University Fukuoka Japan; ^9^ Division of Endoscopy Shizuoka Cancer Center Shizuoka Japan; ^10^ Department of Gastroenterology Tonan Hospital Hokkaido Japan; ^11^ Department of Anesthesiology, Fujimoto General Hospital Miyazaki Japan

**Keywords:** benzodiazepine, elderly, gastrointestinal endoscopy, safety, sedative

## Abstract

**Objectives:**

In gastroenterology, sedation demand is increasing, although elderly patients are more prone to experiencing adverse events. Remimazolam, a novel ultra‐short‐acting benzodiazepine, may reduce recovery time after endoscopic procedures.

**Methods:**

This study was a secondary analysis of the investigator‐initiated trial, which investigated the efficacy and safety of remimazolam in gastrointestinal endoscopy (REM‐IICT JP01). Remimazolam sedation was administered during upper gastrointestinal endoscopy. Patients were divided into two groups: 45 non‐elderly and 11 elderly patients (aged ≥65 years). The primary outcome was sedation success. Secondary outcomes included the dose required for sedation, time to awakening, time to regain the ability to walk, and occurrence of adverse events.

**Results:**

Endoscopic sedation was successful in 95.6% of the non‐elderly group and 100% of the elderly group. The total dose of remimazolam was significantly higher in the non‐elderly group (4.0 [3.0–8.0] mg) than in the elderly group (3.0 [2.0–3.0] mg; *p* < 0.01). The time to awakening was 0.0 (0.0–10.0) min in the non‐elderly group compared to 0.0 (0.0–30.0) min (*p* = 0.98) in the elderly group. The time to regain the ability to walk was significantly longer in the elderly group (5.0 [0.0–60.0] min) than in the non‐elderly group (5.0 [0.0–30.0] min; *p* = 0.03). During the procedure, adverse events included hypotension in two cases (4.4%) in the non‐elderly group and hypoxemia in one case (9.0%) in the elderly group.

**Conclusions:**

Upper gastrointestinal endoscopy with remimazolam was effective and safe, regardless of age.

## INTRODUCTION

During upper gastrointestinal endoscopy without sedation, patients often experience discomfort, leading to a high demand for sedatives.^1^ However, sedatives can potentially cause complications, such as hypoxemia and hypotension, during the procedure. The World Health Organization defines the elderly as individuals aged ≥65 years. Compared to non‐elderly patients, elderly patients are more likely to have comorbidities and experience age‐related declines in heart, lung, kidney, and liver functions, which increases the risk of complications associated with sedation. Particular attention must be given to hypotension, hypoxemia, and aspiration in the elderly compared to younger individuals.^2^

Additionally, the elderly are known to be more sensitive to sedatives,^3^ necessitating the avoidance of overdosing and the implementation of more cautious sedation management. As the population in developed countries ages, the use of anesthesia in elderly patients is expected to increase. Selecting short‐acting sedatives could help prevent prolonged postoperative hypoxemia and shorten recovery time.

Remimazolam, a novel ultra‐short‐acting benzodiazepine, exerts anesthetic and sedative effects by acting on the benzodiazepine‐binding site of gamma‐aminobutyric acid A receptors. It has an ester structure that is rapidly metabolized primarily by liver carboxylesterases.^4^, ^5^, ^6^, ^7^ Flumazenil has a shorter half‐life than other benzodiazepines. Despite numerous recent reports on the usefulness of remimazolam in the field of gastrointestinal endoscopy,^8^, ^9^, ^10^, ^11^, ^12^, ^13^, ^14^ it is only approved for use under general anesthesia in Japan. To enable safer and more reliable sedation during gastrointestinal endoscopy in Japan, we planned an investigator‐initiated clinical trial to investigate the use of remimazolam during gastrointestinal endoscopy. We collaborated with the Pharmaceuticals and Medical Devices Agency of Japan to reach an agreement on the protocol.

We reported the usefulness of remimazolam in the REM‐IICT JP01 trial, an investigator‐initiated clinical trial on endoscopic sedation using remimazolam.^15^, ^16^, ^17^ However, no studies in Japan have demonstrated the effectiveness and safety of remimazolam for elderly patients undergoing upper gastrointestinal endoscopy. Therefore, we conducted a comparative analysis between elderly and non‐elderly patients as a secondary analysis of the REM‐IICT JP01 trial, focusing on the effectiveness and safety of remimazolam in upper gastrointestinal endoscopy.

## METHODS

### Study design

This study was a secondary analysis of an investigator‐initiated trial (REM‐IICT JP01 trial) conducted at seven facilities in Japan, including two clinics. The main study protocol, including this secondary analysis, was approved by the Institutional Review Board of each participating institution. All patients provided written informed consent before enrollment. The main study was registered in the Japan Registry of Clinical Trails (jRCT2031200360).

**FIGURE 1:**
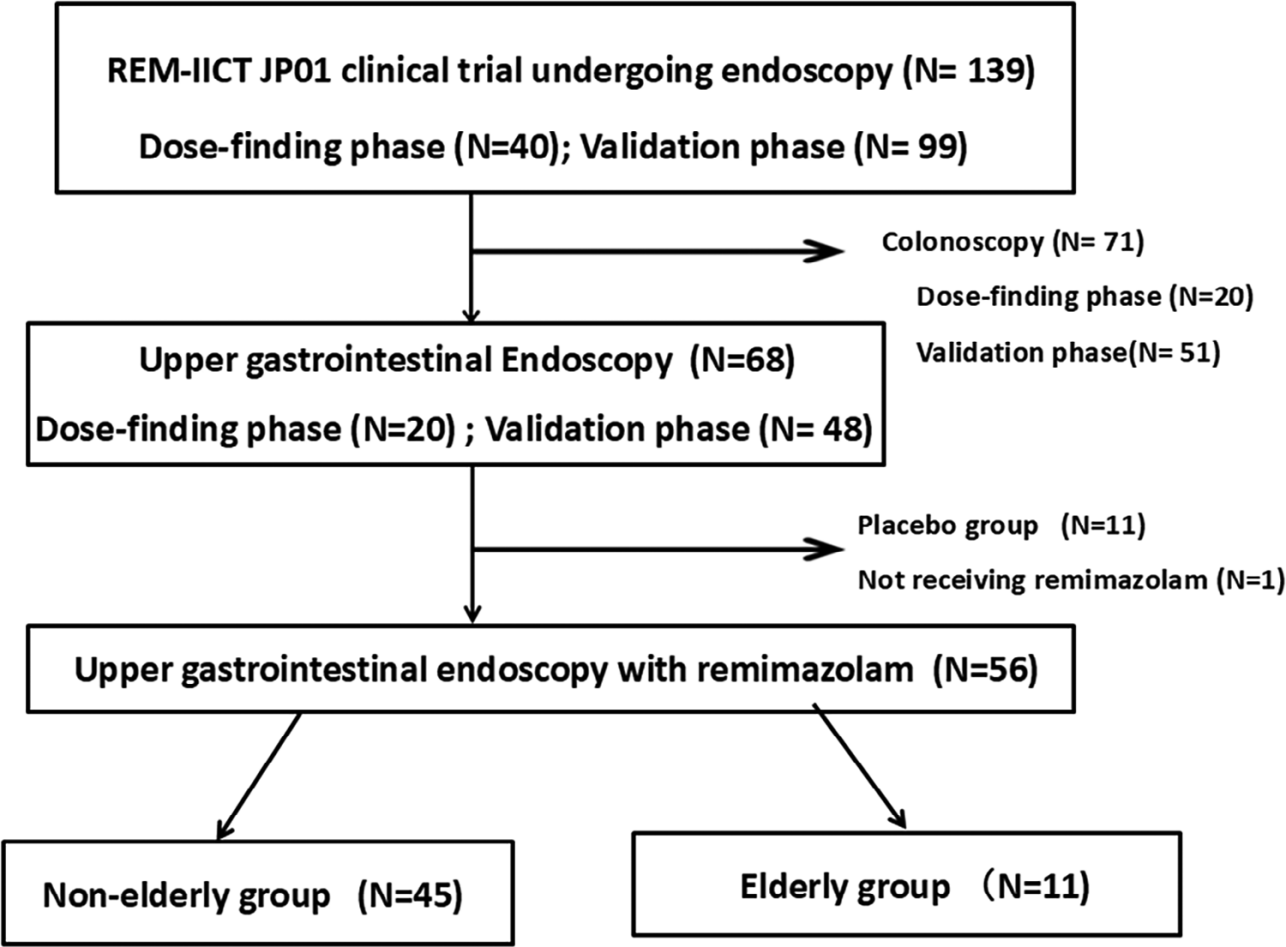
Flowchart of this study.

A flowchart of this study is shown in Figure 1. The REM‐IICT JP01 trial was conducted between April 2021 and December 2021. This study comprised a dose‐finding phase^15^ and a validation phase.^16^ The dose‐finding phase was a single‐arm study with remimazolam, while the validation phase was a placebo‐controlled comparative trial. Regarding patients undergoing upper gastrointestinal endoscopy, 20 patients were included in the dose‐finding phase, and 48 patients were assigned to the remimazolam group in the validation phase. Of these 68 patients, 11 patients were included in the placebo group and one patient did not receive remimazolam treatment, resulting in 56 patients being included in the analysis. These 56 patients undergoing upper gastrointestinal endoscopy and remimazolam administration were divided into two groups: 11 patients in the elderly group (aged ≥65 years) and 45 in the non‐elderly group.

The World Health Organization defines elderly as individuals aged 65 years or older. Furthermore, previous studies on remimazolam have used 65 years as the threshold for defining the elderly group in comparative analyses.^11^, ^18^, ^19^, ^20^ Therefore, in this study, we adopted the definition of elderly as individuals aged 65 years or older.

### Inclusion and exclusion criteria

All participants provided informed consent, with eligibility confirmed through interviews and clinical examinations. The main inclusion criteria were as follows:

Japanese patients scheduled for upper gastrointestinal endoscopy without the use of analgesics.

#### Dose‐finding phase

1. Aged 20–74 years at the time consent was obtained
2. American Society of Anesthesiologists classification of I or II
3. Weight ≥45 kg and ≤70 kg, body mass index <30 kg/m^2^

#### Validation phase

1. The age at the time of obtaining consent is 20 years or older
2. Body mass index is less than 30 kg/m^2^

Furthermore, participants were excluded from the trial if they met any of the following criteria:

1. Heavy alcohol consumption
2. Severe respiratory disease
3. History of sleep apnea
4. Severe liver dysfunction
5. Pregnant or breastfeeding women
6. Any other condition that the physician deemed inappropriate for inclusion in the trial.

### Study procedure and drug administration

Pre‐endoscopy preparation followed standard procedures at each participating medical facility. Analgesics were not administered. Oxygen was not administered during sedation, except when oxygen saturation (SpO_2_) levels dropped, in which case 2–5 L of oxygen was provided. An initial dose of 2 or 3 mg of remimazolam was administered intravenously. In elderly patients, the dose can be reduced by half at the discretion of the endoscopist. Sedation levels were assessed at 2‐min intervals using the MOAA/S score^21^ from the start of initial administration. Once sedation (MOAA/S score of ≤4) was achieved, the gastrointestinal endoscopy began. If the patient was awake (MOAA/S score 5), 1 mg of remimazolam was administered slowly. Rescue medications such as midazolam or propofol were not allowed. After the start of the endoscopy, vital signs (blood pressure, heart rate, and respiratory rate), SpO_2_, and MOAA/S scores were measured at 5‐min intervals. After endoscopy, vital signs (blood pressure, heart rate, and respiratory rate), SpO_2_, and MOAA/S scores were evaluated at the time of completion, at 5 min, and every 10 min until 60 min post‐procedure. Once the MOAA/S score reached 5, the patient's walking ability was assessed. Walking was considered possible if the patient could walk a 5‐m straight line without staggering. The attending physician conducted an interview 7–14 days after the endoscopy to check for any adverse events that occurred after discharge.

### Outcomes

The primary outcome was defined as the success rate of sedation in gastrointestinal endoscopy, evaluated based on the following three criteria:

1. Achieving sedation (MOAA/S score of ≤4) before the start of endoscopy.
2. Completion of the gastrointestinal endoscopy.
3. The number of additional doses administered after the start of endoscopy did not exceed two doses per 6 min.

The primary endpoint was determined based on a previous report and the procedure time of upper gastrointestinal endoscopy.^9^

Secondary outcomes included the dosage required to achieve sedation, time from the end of the procedure to awakening, time from the end of the procedure to regaining walking ability, and occurrence of adverse events. In addition, a questionnaire survey was administered to both physicians and patients regarding their satisfaction with the procedure. The survey was evaluated on a 5‐point scale: 5 = Very Satisfied, 4 = Satisfied, 3 = Neutral, 2 = Somewhat Dissatisfied, and 1 = Very Dissatisfied.

### Definition

Completion of upper gastrointestinal endoscopy was defined as the successful observation of four specific areas across three organs: the esophagus, stomach (including the body of the stomach observed both in the forward and retroflexed views), and duodenum (up to the descending part). Oxygen administration was initiated when SpO2 dropped below 94%, at 2–5 L/min. If the respiratory rate fell to 5 bpm or fewer, airway management was performed. If breathing did not recover despite airway management, manual ventilation using a bag‐valve mask or similar device was administered. Loss of consciousness was defined as a MOAA/S score of ≤1 at two consecutive time points. Flumazenil was administered intravenously only if urgently needed to reverse sedation from the investigational drug. Adverse events severity was assessed according to Common Terminology Criteria for Adverse Events v5.0.

### Statistical analysis

All continuous variables were expressed as medians (range). Categorical variables were compared between the groups using Fisher's exact test, and continuous variables were compared using the Wilcoxon rank‐sum test. Statistical significance was set at *p* < 0.05. All statistical analyses were performed using JMP software (version 13.0.0; SAS Institute).

## RESULTS

Patients were divided into two groups: 45 non‐elderly and 11 elderly patients. Three were aged ≥ 75 years and dose reduction was applied to only one of these patients.

### Baseline characteristics

Baseline characteristics are shown in Table 1. No significant differences were observed between the two groups regarding sex, height, weight, or body mass index. However, the proportion of patients classified as American Society of Anesthesiologists II or III was higher in the elderly group compared to the non‐elderly group.

**TABLE 1:** Baseline characteristics.

	Non‐elderly group, *n* = 45	Elderly group, *n* = 11	*p*‐value
Age, (range)	47.0 (22.0–64.0)	73.0 (69.0–84.0)	<0.01
Male/female, *n* (%)	17 (37.8%)/28 (62.2%)	6 (54.5%)/5 (45.5%)	0.33
Height, cm (range)	160.4 (147.2–182.0)	158.9 (147.0–177.8)	0.14
Weight, kg (range)	55.5 (38.7–77.6)	52.5 (40.4–72.4)	0.24
BMI, kg/m^2^ (range)	21.2 (15.6–29.9)	20.9 (18.7–25.7)	0.80
ASA (I/II/ III), *n* (%)	34 (75.6%)/11 (24.4%)/0 (0%)	3 (27.2%)/7 (63.6%)/1 (9.1%)	<0.01

*Note*: Data are presented as median (range) or number (percentage).

Abbreviations: ASA, American Society of Anesthesiologists classification; BMI, body mass index.

### Efficacy assessment of sedation

Table 2 compares the efficacy assessment of remimazolam between two groups. The success rate of sedation, which was the primary outcome, was 95.6% (43/45) in the non‐elderly group and 100% (11/11) in the elderly group, with no significant intergroup differences between the two groups. The initial dose of remimazolam was 3.0 mg in both groups, with no significant difference. However, the doses administered before the start of the procedure were significantly higher in the non‐elderly group compared to the elderly group: 3.0 (2.0–8.0) mg vs. 3.0 (2.0–4.0) mg before the procedure, 1.0 (0.0–2.0) mg vs. 0.0 (0.0–1.0) mg after the start of the procedure, and 4.0 (3.0–8.0) mg vs. 3.0 (2.0–4.0) mg for the total dose, all showing significantly higher amounts in the non‐elderly group (*p* < 0.01). The time from the end of the procedure to awakening was 0.0 min for both the non‐elderly and elderly groups, with no significant difference. However, the time from the end of the procedure to the ability to walk was significantly longer in the elderly group compared to the non‐elderly group: 5.0 (0.0–30.0) min vs. 5.0 (5.0–60.0) min (*p* = 0.03).

**TABLE 2:** Comparison of the effectiveness of sedatives between the two groups

	Non‐elderly group, *n* = 45	Elderly group, *n* = 11	*p*‐value
Successful sedation	43 (95.6%)	11 (100.0%)	1.0
Initial dose, mg (range)	3.0 (2.0–3.0)	3.0 (1.5–3.0)	1.0
Patients with reduced dosage, *n* (%)	0 (0.0%)	1 (9.1%)	1.0
Total dose of the before procedure, mg (range)	3.0 (2.0–8.0)	3.0 (2.0–4.0)	<0.01
Additional dose after procedure, mg (range)	1.0 (0.0–2.0)	0.0 (0.0–1.0)	<0.01
Total dose, mg (range)	4.0 (3.0–8.0)	3.0 (2.0–4.0)	< 0.01
Procedure time, min (range)	6.0 (4.0–10.0)	7.0 (4.0–13.0)	0.09
Time from end of procedure to awaking, min (range)	0.0 (0.0–30.0)	0.0 (0.0–10.0)	0.98
Time from end of procedure to regain the ability to walk, min (range)	5.0 (0.0–30.0)	5.0 (0.0–60.0)	0.03

*Note*: Data are presented as median (range) or number (percentage).

### Safety assessments of sedation

The adverse events are shown in Table 3. Adverse events during upper gastrointestinal endoscopy were observed in two cases (4.4%) of hypotension in the non‐elderly group and in one case (9.1%) of hypoxemia in the elderly group. No patients required flumazenil or manual ventilation. Both groups experienced minor post‐procedural complications, with no severe complications reported in any of the cases.

**TABLE 3:** Safety assessment.

Adverse event grade (I/II/III‐V), *n* (%)	Non‐elderly group, *n* = 45	Elderly group, *n* = 11
During endoscopy		
Hypotension	2 (4.4)/0 (0.0)/0 (0.0)	0 (0.0)/0 (0.0)/0 (0.0)
Hypoxemia	0 (0.0)/0 (0.0)/0 (0.0)	1 (9.1)/0 (0.0)/0 (0.0)
After endoscopy		
Drowsiness	1 (2.2)/1 (2.2)/0 (0.0)	0 (0.0)/0 (0.0)/0 (0.0)
Head discomfort/headache	2 (4.4)/2 (4.4)/0 (0.0)	0 (0.0)/0 (0.0)/0 (0.0)
Fatigue	1 (2.2)/0 (0.0)/0 (0.0)	0 (0.0)/0 (0.0)/0 (0.0)
Abdominal discomfort/abdominal pain	2 (4.4)/0 (0.0)/0 (0.0)	0 (0.0)/0 (0.0)/0 (0.0)
Sore throat	1 (2.2)/0 (0.0)/0 (0.0)	0 (0.0)/0 (0.0)/0 (0.0)
Dizziness	0 (0.0)/0 (0.0)/0 (0.0)	1 (9.1)/0 (0.0)/0 (0.0)

*Note*: Data are presented as numbers (percentages).

### Questionnaire

The results of the physician and patient satisfaction surveys are presented in Tables 4 and 5. No significant differences were observed between the two groups regarding the time required to achieve sedation, depth of sedation, pain relief, or satisfaction levels of physicians and patients.

**TABLE 4:** Physician satisfaction.

	Non‐elderly group, *n* = 45	Elderly group, *n* = 11	*p*‐value
Time to sedation (1–2/3/4–5), *n* (%)	0 (0.0)/2 (4.4)/43 (95.6)	0 (0.0)/1 (9.1)/10 (90.9)	0.74
Ease of procedure (1–2/3/4–5), *n* (%)	0 (0.0)/1 (2.2)/44 (97.8)	0 (0.0)/0 (0.0)/11 (100.0)	0.89
Sedation depth intraoperatively (1–2/3/4–5), *n* (%)	0 (0.0)/3 (6.7)/42 (93.3)	0 (0.0)/1 (9.1)/10 (90.9)	0.39
Time to regain consciousness after the procedure (1–2/3/4–5), *n* (%)	0 (0.0)/2 (4.4)/43 (95.6)	0 (0.0)/0 (0.0)/11 (100.0)	0.31
Patient satisfaction reported by the physician (1–2/3/4–5), *n* (%)	0 (0.0)/2 (4.4)/43 (95.6)	0 (0.0)/0 (0.0)/11 (100.0)	0.15
Willingness to use the drugs again (1–2/3/4–5), *n* (%)	0 (0.0)/0 (0.0)/45 (100.0)	0 (0.0)/0 (0.0)/11 (100.0)	1.0

*Note*: Data are presented as numbers (percentages).

**TABLE 5:** Patient satisfaction.

	Non‐elderly group, *n* = 45	Elderly group, *n* = 11	*p*‐value
Time to sedation (1–2/3/4–5), *n* (%)	2 (4.4)/4 (8.9)/39 (86.7)	0 (0.0)/0 (0.0)/11 (100.0)	0.38
Pain reduction (1–2/3/4–5), *n* (%)	2 (4.4)/1 (2.2)/42 (93.4)	1 (9.1)/0 (0.0)/10 (90.9)	0.72
Sedation depth intraoperatively (1–2/3/4–5), *n* (%)	4 (8.9)/4 (8.9)/37 (82.2)	0 (0.0)/1 (9.1)/10 (90.9)	0.92
Time to regaining consciousness postoperatively (1–2/3/4–5), *n* (%)	0 (0.0)/2 (4.4)/43 (95.6)	0 (0.0)/1 (9.1)/10 (90.9)	0.24
Willingness to use drugs again (1–2/3/4–5), *n* (%)	1 (2.2)/3 (6.7)/41 (91.1)	0 (0.0)/3 (27.3)/8 (72.7)	0.09

*Note*: Data are presented as numbers (percentages).

## DISCUSSION

We conducted a comparative study of the effectiveness and safety of remimazolam in elderly and non‐elderly Japanese patients undergoing upper gastrointestinal endoscopy. Although reports exist on remimazolam use in elderly patients,^18^, ^19^, ^20^, ^22^, ^23^, ^24^ to our knowledge, this is the first study to provide data specific to Japanese patients. Unlike in other countries, analgesics are rarely used in combination with sedatives during upper gastrointestinal endoscopy in Japan. Therefore, this study is one of the few to use remimazolam alone for sedation.

The results showed that sedation with remimazolam was effective in most cases, regardless of whether the patient was elderly or not. A distinctive feature of this study was the lower dosage of remimazolam used compared to previous reports. Other studies have used an initial dose of 0.10–0.20 mg/kg (approximately 6–12 mg) of remimazolam. These studies aimed for deep sedation, starting with a target MOAA/S score of 1–3 or lower.^21^, ^22^, ^23^ However, guidelines in the field of gastrointestinal endoscopy emphasize the importance of moderate sedation (MOAA/S score 3 or 4) rather than deep sedation, as the latter increases the risk of adverse events.

Reports suggest that high doses of remimazolam (0.20 mg/kg) in elderly patients may lead to a decline in cognitive function (immediate and short‐delay recall) after upper gastrointestinal endoscopy.^23^ In our study, no cases of loss of consciousness (MOAA/S score of ≤1) or the need for flumazenil occurred. Additionally, elderly patients did not require additional doses compared with non‐elderly patients. In the questionnaire survey, most patients reported a reduction in discomfort, and the endoscopists were highly satisfied. Considering these findings, an initial dose of 3 mg appears to be ideal for upper gastrointestinal endoscopy.

Only a few reports have compared recovery times between elderly and non‐elderly patients using remimazolam as a sedative during upper gastrointestinal endoscopy. Our study found no significant difference in the time to awakening after the procedure; however, elderly patients required significantly more time to regain walking ability. This suggests that even with remimazolam, which has a short half‐life, more cautious post‐procedural observation is necessary for elderly patients.

From a safety perspective, remimazolam has been reported to cause fewer adverse events, such as hypoxemia and hypotension, in elderly patients compared to propofol.^18^, ^19^, ^20^ A meta‐analysis that included seven randomized controlled trials with a total of 1466 patients (731 treated with remimazolam and 735 treated with propofol) found that remimazolam was significantly associated with lower rates of bradycardia (*p* = 0.02, five studies, 1323 patients) and hypoxemia (*p* < 0.00001, six studies, 1389 patients), although propofol demonstrated a significantly shorter time to loss of consciousness (*p* < 0.01) and higher sedation success after the first dose (*p* = 0.05).^23^ In our study, only two cases of hypotension (4.4%) and one case of hypoxemia (9.0%) were observed in the non‐elderly and elderly groups, respectively. Remimazolam, which is safer for use in elderly patients and has an available antagonist, might be a more suitable drug where the shortage of anesthesiologists limits the use of propofol. We also investigated the efficacy and safety of remimazolam in therapeutic procedures, such as endoscopic submucosal dissection, biliary and pancreatic endoscopy, and enteroscopy, and have been collecting cases (jRCT2031220129). We believe that remimazolam has the potential to be widely used in examinations as well as in therapeutic applications.

Remimazolam has its shortcomings. There is some uncertainty regarding the compatibility of remimazolam with Bispectral Index monitoring for assessing the depth of anesthesia, unlike propofol. This lack of reliable monitoring for anesthesia depth is considered a limitation in the clinical application of remimazolam. Compared to midazolam, remimazolam offers the advantage of faster recovery. However, this can lead to the need for more frequent additional doses during prolonged procedures or treatments, which may pose a challenge in maintaining consistent sedation over extended periods.

This study has several strengths. First, this was a multicenter collaborative study. Second, by not using analgesics or allowing rescue medications, we obtained data free of interactions with other drugs in both groups.

However, this study has several limitations. First, the study population was limited to Japanese patients, excluding those with conditions such as liver dysfunction. These results may vary among different populations, particularly among elderly patients from different ethnic backgrounds or those with comorbidities. Second, the sample size was relatively small. Additionally, this study was not a randomized controlled trial and did not match baseline American Society of Anesthesiologists classifications between non‐elderly and elderly patients. Third, the dose‐finding and validation phases had differing eligibility criteria based solely on age and weight; however, both phases were included in this study. Fourth, there was a discrepancy between the definition of extremely elderly individuals for whom the dose reduction of remimazolam could be considered and the criteria for elderly individuals employed in this study. Therefore, further prospective, comparative studies are warranted.

In conclusion, remimazolam is effective without severe adverse events for elderly patients undergoing upper gastrointestinal endoscopy. Further accumulation of cases and the collection of more detailed data are necessary in the future.

## CONFLICT OF INTEREST STATEMENT

Hisatomo Ikehara received honoraria for his lectures from FUJIFILM Corporation, Mitsubishi Tanabe Pharma Corporation, and Olympus Corporation.

## ETHICS STATEMENT

The main study protocol (REM‐IICT‐JP01) was approved by the Institutional Review Board of each participating institution. All patients provided written informed consent before enrollment. The main study was registered in the Japan Registry of Clinical Trails (jRCT2031200360).

## Data Availability

The data that support the findings of this study are not publicly available as they contain information that could compromise the privacy of research participants but are available from the corresponding author Hisatomo Ikehara upon reasonable request.
